# Booster dose of COVID-19 mRNA vaccine does not increase risks of myocarditis and pericarditis compared with primary vaccination: New insights from the vaccine adverse event reporting system

**DOI:** 10.3389/fimmu.2022.938322

**Published:** 2022-09-12

**Authors:** Congqin Chen, Fang Fu, Lingqing Ding, Jie Fang, Jie Xiao

**Affiliations:** ^1^ Department of Pharmacy, Xiamen Cardiovascular Hospital of Xiamen University, School of Medicine, Xiamen University, Xiamen, China; ^2^ Department of Pharmacy, Ruijin Hospital, School of Medicine, Shanghai Jiaotong University, Shanghai, China

**Keywords:** myocarditis, pericarditis, COVID-19 mRNA vaccination, booster dose, primary series

## Abstract

**Background:**

Despite the likely association between coronavirus 2019 (COVID-19) mRNA vaccines and cases of myocarditis/pericarditis, the benefit–risk assessment by the Centers for Disease Control (CDC) still showed a favorable balance for the primary series of COVID-19 mRNA vaccinations. Since August 2021, a full-scale booster vaccination in certain recipients has been recommended. Great concerns about whether the COVID-19 mRNA booster vaccination could increase the risks of myocarditis/pericarditis have been raised since then. The present study aimed to compare the incidence rates and risks of myocarditis/pericarditis between booster and primary vaccination programs.

**Methods:**

The CDC COVID Data Tracker and the Vaccines Adverse Event Reporting System (VAERS) were queried between December 11, 2020 and March 15, 2022. Incidence rates were calculated by cases of myocarditis/pericarditis divided by the number of vaccinated people or the total doses of COVID-19 mRNA vaccines. Disproportionality patterns for myocarditis/pericarditis of different COVID-19 mRNA vaccinations were accessed based on the reporting odds and proportional reporting ratios (ROR and PRR, respectively).

**Results:**

A total of 2,588 reports of myocarditis/pericarditis were identified after administration of primary-series COVID-19 mRNA vaccination and 269 after the booster dose program during the study period. The incidence of myocarditis/pericarditis following booster COVID-19 mRNA vaccination was lower than that of primary series. The results showed significantly high reporting of myocarditis/pericarditis following the administration of primary COVID-19 mRNA vaccination, whereas the disproportional level was lower in the booster-dose vaccination.

**Conclusion:**

This study found that the booster dose of COVID-19 mRNA vaccination when compared with primary series course did not lead to an increase in the risks of myocarditis/pericarditis.

## Introduction

In the United States (US), two messenger RNA (mRNA) coronavirus disease 2019 (COVID-19) vaccines, BNT162b2 mRNA (Pfizer-BioNTech) and mRNA-1273 (Moderna) were granted emergency use authorization (EUA) by the US Food and Drug Administration (FDA) in December 2020 (1, 2). With the increasing number of administered COVID-19 mRNA vaccines, growing evidence for myocarditis and pericarditis as rare complications after COVID-19 mRNA vaccination has been reported, especially in young adult and adolescent males (3–10). Recently, the Advisory Committee on Immunization Practices (ACIP) of Centers for Disease Control (CDC) identified a likely association between COVID-19 mRNA vaccines and cases of myocarditis/pericarditis (11). The ACIP also conducted a benefit–risk assessment for COVID-19 mRNA vaccination and myocarditis/pericarditis with the conclusion that the benefits outweigh the risks, and the continued use of mRNA COVID-19 vaccines is still recommended for all age groups (11).

Since August 2021, a series of EUAs and recommendations, including those for an additional primary dose for immunocompromised persons and a booster dose for persons having been vaccinated with two doses of mRNA vaccine, were approved because of reduced immunogenicity in immunocompromised persons, waning vaccine effectiveness over time, and the introduction of the highly transmissible variants (12). The recommendation was extended to certain recipients aged ≥ 12 years soon afterwards, starting a full-scale booster campaign (13). Several myocarditis/pericarditis cases following booster shot of mRNA COVID-19 vaccines have been published (14–17). Concerns about whether the favorable balance of primary series still stand for booster dose and whether the booster vaccination could lead to an increase in the risks of myocarditis/pericarditis have been raised in addition the hesitancy to receive a booster dose.

No population-based study addressing these doubts has been performed to date. Thus, to further evaluate whether the booster dose of COVID-19 mRNA vaccine when compared with the primary series could cause an increase in the risks of myocarditis/pericarditis, a population-based study based on the CDC COVID Data Tracker and the Vaccines Adverse Event Reporting System (VAERS) was conducted to investigate the incidence rates and risks of myocarditis/pericarditis following the booster dose and primary series of COVID-19 mRNA vaccinations.

## Methods

### Data source

VAERS is a US system for reporting adverse events following immunization (AEFIs) that is co-administered by the CDC and the FDA (18, 19). VAERS accepts reports from vaccine manufacturers, health-care providers, vaccine recipients, and others. The VAERS reports include information concerning age, sex, administered vaccines, dose and lot number, post-vaccination adverse events (AEs), and health history. Signs and symptoms of AEs are coded by trained personnel using the Medical Dictionary for Regulatory Activities (MedDRA), a clinically validated, internationally standardized terminology (19, 20). VAERS can be applied to detect unexpected patterns of AEFIs which are unlikely to be detected in clinical trials because of the limited number of participating vaccine recipients (21–25). The CDC COVID Data Tracker is another data source used in this study. It provides comprehensive information on COVID-19 vaccinations in the US, including delivered and administered doses, the number of people who received at least one dose, number of people who are fully vaccinated, and number of people who received booster dose based on vaccine type (26).

### Data extraction

VAERS data (from December 11, 2020 to March 15, 2022) were downloaded from the FDA website. Raw VAERS data were managed locally using the Microsoft Access software (version 2021 X32). Each report was classified based on the following binomial factors: (1) “with” or “without” exposure to the administration of vaccines of interest (namely, BNT162b2 from Pfizer-BioNTech and mRNA-1273 from Moderna) and (2) “with” or “without” the development of an AEFI category of interest, which was defined by combining the MedDRA 24.1 preferred terms (PTs) of “myocarditis”, “pericarditis”, and “myopericarditis”. Each narrative myocarditis/pericarditis report and laboratory results were reviewed. Data, such as age, sex, dose, seriousness, and AE onset interval (from vaccination date [day 0] to the reported onset of first symptoms) were also collected. The total doses of COVID-19 mRNA vaccine by administered and number of people vaccinated through different vaccination programs were acquired through the CDC COVID Data Tracker. According to the Tracker, this study counts people as having “received a booster dose” if they are fully vaccinated and received another dose of any COVID-19 mRNA vaccine on or after August 13, 2021. This system does not distinguish whether the recipient is immunocompromised or received an additional dose.

### Data analysis

The incidence rate of myocarditis/pericarditis was estimated using reports of myocarditis/pericarditis divided by the number of vaccinated people or the total doses of administered COVID-19 mRNA vaccines during the same study period. A population-based pharmacovigilance analysis using a case/non-case approach was performed to access the risks of myocarditis/pericarditis after different COVID-19 mRNA vaccination programs. This system is a common approach used in pharmacovigilance studies to identify safety signals (22, 23, 27). From the mathematical point of view, the idea of the case/non-case approach is to compare the proportion of an AE of interest in people exposed to a specific vaccine (cases) with the reports of the same reaction in people who were not exposed to this vaccine (non-cases) (28, 29). This so-called case/non-case approach can be considered a case-control analysis, and results can be measured using the reporting odds ratio and proportional reporting ratio (ROR and PRR, respectively) with their 95% confidence interval (95% CI) (30). The signal was defined by the criterion of the lower limit of the 95% CI of ROR or PRR > 1. The higher the 95%CI is, the more significant the signal is.

**Figure 1:**
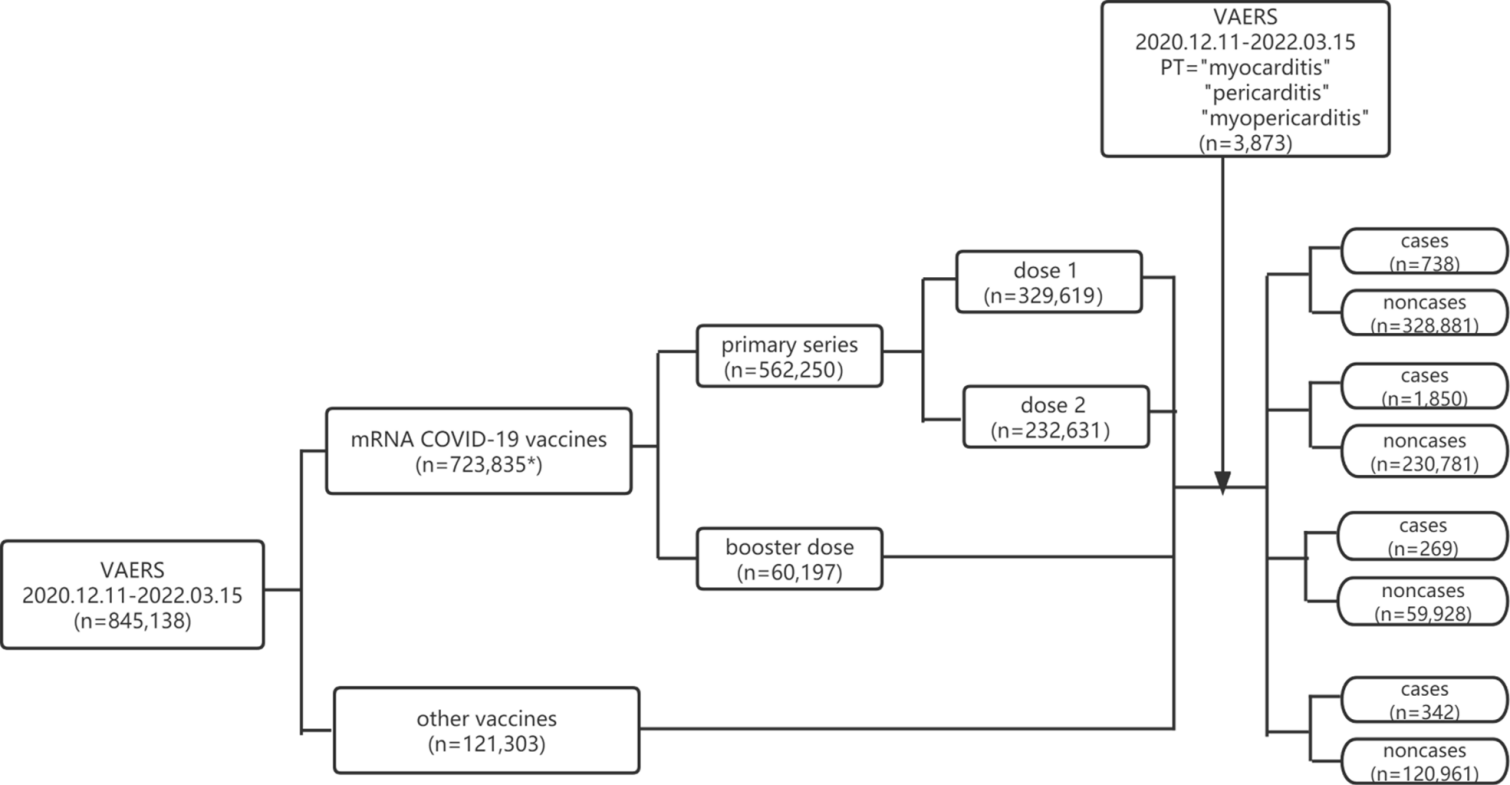
Flowchart of identifying cases and non-cases from VAERS database. VAERS, Vaccine Adverse Event Reporting System; mRNA, messenger RNA; COVID-19, coronavirus disease 2019; PT, preferred term. * including reports of primary series, booster dose and unspecified dose.

In our study, disproportionality was accessed by ROR and PRR. ROR= (Na * Nd)/(Nb * Nc) and PRR= (Na/[Na+Nb])/(Nc/[Nc+Nd]) in which Na is the number of reports of myocarditis/pericarditis for COVID-19 mRNA vaccines (primary series or booster dose), Nb represents the reports for COVID-19 mRNA vaccines (primary series or booster dose) without reporting myocarditis/pericarditis, Nc is the number of the reports of myocarditis/pericarditis for all other vaccines, Nd represents the number of the reports for all other vaccines without reporting myocarditis/pericarditis. The flowchart for identifying cases and non-cases from the VAERS database is shown in **Figure 1**.

## Results

### Incidence of COVID-19 mRNA vaccine related myocarditis/pericarditis

As of March 15, 2022, a total of 538,368,518 doses of COVID-19 mRNA vaccine (443,705,709 doses for primary series and 94,662,809 doses for booster shot) were administered, and 199,808,526 people have been fully vaccinated with COVID-19 mRNA vaccine in the US. Overall, 723,835 AEFIs were found to be related to COVID-19 mRNA vaccines, and 3,873 reports related to myocarditis/pericarditis were documented in the VAERS database during the analytical period. Among them, the primary-series mRNA COVID-19 vaccination was identified as the suspected vaccine causing myocarditis/pericarditis in 2,588 reports, while booster-dose vaccination in 269 reports. The incidences of myocarditis/pericarditis are summarized in **Tables 1**, **2**. AEs of myocarditis/pericarditis following primary-series COVID-19 mRNA vaccination were rare with an overall incidence rate of 5.83 (95% CI 5.61–6.06) cases per million doses administered and 10.61 (95% CI 10.21–11.03) cases per million vaccine recipients. The incidence rate for the booster dose was 2.84 (95% CI 2.51–3.20), which was significantly lower than the primary series. Among the primary series, the incidence rate was much higher for dose 2 at 9.26 (95% CI 8.84–9.69), and it decreased for dose 1 to 3.03 (95% CI 2.81–3.25). Stratified analyses by manufacturer were carried out. The results showed that the incidence rates of the booster dose were significantly lower than the primary series for both Pfizer-BioNTech and Moderna.

**Table 1:** The incidence rate of cases of myocarditis/pericarditis by different COVID-19 mRNA vaccination programs based on total administered dose.

Manufacturer	Vaccination program		Total doses administered	Myocarditis		Pericarditis		Myopericarditis		In total	
				Cases	Incidence rate*	Cases	Incidence rate*	Cases	Incidence rate*	Cases	Incidence rate*
**Pfizer-BioNTech**	**Primary series**										
		**Dose 1**	151,453,547	189	1.25 (1.08-1.44)^#^	185	1.22 (1.05-1.41)^#^	33	0.22 (0.15-0.31)^#^	407	2.69 (2.43-2.96)^#^
		**Dose 2**	124,212,958	773	6.22 (5.79-6.68)	395	3.18 (2.87-3.51)	107	0.86 (0.71-1.04)	1,275	10.26 (9.71-10.84)
		**In total**	275,666,505	962	3.49 (3.27-3.72)	580	2.1 (1.94-2.28)	140	0.51 (0.43-0.60)	1,682	6.10 (5.81-6.40)
	**Booster dose**										
		**Dose 3**	53,149,023	78	1.47 (1.16-1.83)^#%^	71	1.34 (1.04-1.69)^#%^	10	0.19 (0.09-0.35)^#%^	159	3.00 (2.55-3.49)^#%^
**Moderna**	**Primary series**										
		**Dose 1**	92,443,636	181	1.96 (1.68-2.27)^#^	124	1.34 (1.12-1.60)^#^	26	0.28 (0.18-0.41)^#^	331	3.58 (3.21-3.99)^#^
		**Dose 2**	75,595,568	293	3.88 (3.45-4.35)	225	2.98 (2.60-3.40)	57	0.75 (0.57-0.98)	575	7.61 (7.00-8.25)
		**In total**	168,039,204	474	2.82 (2.57-3.09)	349	2.08 (1.87-2.31)	83	0.49 (0.39-0.61)	906	5.39 (5.05-5.75)
	**Booster dose**										
		**Dose 3**	41,513,786	58	1.40 (1.06-1.81)^#%^	46	1.11 (0.81-1.48)^#%^	6	0.14 (0.05-0.31)^#%^	110	2.65 (2.18-3.19)^#%^
**All mRNA**	**Primary series**										
		**Dose 1**	243,897,183	370	1.52 (1.37 - 1.68)^#^	309	1.27 (1.13 - 1.42)^#^	59	0.24 (0.18 - 0.31)^#^	738	3.03 (2.81-3.25)^#^
		**Dose 2**	199,808,526	1,066	5.34 (5.02 - 5.67)	620	3.10 (2.86 - 3.36)	164	0.82 (0.70 - 0.96)	1,850	9.26 (8.84-9.69)
		**In total**	443,705,709	1,436	3.24 (3.07 - 3.41)	929	2.09 (1.96 - 2.23)	223	0.50 (0.44 - 0.57)	2,588	5.83 (5.61-6.06)
	**Booster dose**										
		**Dose 3**	94,662,809	136	1.44 (1.21 - 1.70)^#%^	117	1.24 (1.02 - 1.48)^#%^	16	0.17 (0.09 - 0.27)^#%^	269	2.84 (2.51-3.20)^%#^

*Defined as per million doses (95% confidence interval [95% CI]); ^%^ P < 0.05, compared with primary series; ^#^P < 0.05, compared with dose 2.

mRNA, messenger RNA; COVID-19, coronavirus disease 2019.

**Table 2:** The incidence rate of cases of myocarditis/pericarditis by different mRNA COVID-19 vaccination programs based on number of people vaccinated.

Manufacturer	Vaccination program		Number of people vaccinated	Myocarditis		Pericarditis		Myopericarditis		In total	
				Cases	Incidence rate^@^	Cases	Incidence rate^@^	Cases	Incidence rate^@^	Cases	Incidence rate^@^
**Pfizer-BioNTech**	**Primary series**										
		**Dose 1**	151,453,547	189	1.25 (1.08-1.44)^#^	185	1.22 (1.05-1.41)^#^	33	0.22 (0.15-0.31)^#^	407	2.69 (2.43-2.96)^#^
		**Dose 2**	124,212,958	773	6.22 (5.79-6.68)	395	3.18 (2.87-3.51)	107	0.86 (0.71-1.04)	1,275	10.26 (9.71-10.84)
		**In total**	151,453,547	962	6.35 (5.96-6.77)	580	3.83 (3.52-4.15)	140	0.92 (0.78-1.09)	1,682	11.11 (10.58-11.65)
	**Booster dose**										
		**Dose 3**	53,149,023	78	1.47 (1.16-1.83)^#%^	71	1.34 (1.04-1.69)^#%^	10	0.19 (0.09-0.35)^#%^	159	3.00 (2.55-3.49)^#%^
**Moderna**	**Primary series**										
		**Dose 1**	92,443,636	181	1.96 (1.68-2.27)^#^	124	1.34 (1.12-1.60)^#^	26	0.28 (0.18-0.41)^#^	331	3.58 (3.21-3.99)^#^
		**Dose 2**	75,595,568	293	3.88 (3.45-4.35)	225	2.98 (2.60-3.40)	57	0.75 (0.57-0.98)	575	7.61 (7.00-8.25)
		**In total**	92,443,636	474	5.13 (4.68-5.61)	349	3.78 (3.39-4.19)	83	0.90 (0.72-1.11)	906	9.80 (9.17-10.46)
	**Booster dose**										
		**Dose 3**	41,513,786	58	1.40 (1.06-1.81)^#%^	46	1.11 (0.81-1.48)^#%^	6	0.14 (0.05-0.31)^#%^	110	2.65 (2.18-3.19)^#%^
**All mRNA**	**Primary series**										
		**Dose 1**	243,897,183	370	1.52 (1.37 - 1.68)#	309	1.27 (1.13 - 1.42)#	59	0.24 (0.18 - 0.31)#	738	3.03 (2.81-3.25)#
		**Dose 2**	199,808,526	1,066	5.34 (5.02 - 5.67)	620	3.10 (2.86 - 3.36)	164	0.82 (0.70 - 0.96)	1,850	9.26 (8.84-9.69)
		**In total**	243,897,183	1,436	5.89 (5.59 - 6.20)	929	3.81 (3.57 - 4.06)	223	0.91 (0.80 - 1.04)	2,588	10.61 (10.21-11.03)
	**Booster dose**										
		**Dose 3**	94,662,809	136	1.44 (1.21 - 1.70)%#	117	1.24 (1.02 - 1.48)%#	16	0.17 (0.09 - 0.27)%#	269	2.84 (2.51-3.20)%#

^@^ Defined as per million vaccinees (95% CI); ^%^ P < 0.05, compared with primary series; ^#^P < 0.05, compared with dose 2.

VAERS, Vaccine Adverse Event Reporting System; mRNA, messenger RNA; COVID-19, coronavirus disease 2019..

### Descriptive analysis

The clinical characteristics of these vaccine recipients are presented in **Table 3**. Overall, cases of myocarditis/pericarditis were more common after administration of primary-series COVID-19 vaccination, especially following the second dose. For both primary series and booster dose, cases of myocarditis/pericarditis were more likely to be reported in middle/younger males (age ranging from 25- to 44-year-old). Cases of myocarditis/pericarditis were more likely to be serious than other reported AEs. For primary series, about 60% of AEs of myocarditis/pericarditis were hospitalized, 50.54% had emergency room (ER) visits, and 1.16% died. For reports of myocarditis/pericarditis following booster dose, 48.70% of the cases were hospitalized, 55.90% had ER visits, and 1 out of 269 cases (0.37%) died. For the primary series, myocarditis accounted for 1436 (55.49%) reports, pericarditis accounted for 929 (35.90%) reports, and in 223 (8.62%) reports, patients developed symptoms of myopericarditis. For booster doses, myocarditis still accounted for about 50% of the cases while pericarditis accounted for 43.49% of the cases.

**Table 3:** Characteristics of reports to the VAERS following mRNA COVID-19 vaccination. .

Characteristics		Primary series						Booster dose		*P* value
		Dose 1		Dose 2		In total		Dose 3		
		Cases	Noncases	Cases	Noncases	Cases	Noncases	Cases	Noncases	
n(%)		738	328,881	1,850	230,781	2,588	559,662	269	59,928	0.64
**Sex**										
	Female	300 (40.65)	229,761 (69.86)	505 (27.30)	156,113 (67.65)	805 (31.11)	385,874 (68.95)	110 (40.89)	37,549 (62.66)	< 0.05
	Male	427 (57.86)	88,089 (26.78)	1,329 (71.84)	70,556 (30.57)	1,756 (67.85)	158,645 (28.35)	156 (57.99)	19,966 (33.32)	< 0.05
	Not specified	11 (1.49)	11,031 (3.35)	16 (0.86)	4,112 (1.78)	27 (1.04)	15,143 (2.71)	3 (1.12)	2,413 (4.03)	
**Age (y)**										
	<18	81 (10.98)	20,591 (6.26)	387 (20.92)	10,606 (4.60)	468 (18.08)	31,197 (5.57)	18 (6.69)	2,275 (3.80)	< 0.05
	18-24	114 (15.45)	16,765 (5.10)	392 (21.19)	11,054 (4.79)	506 (19.55)	27,819 (4.97)	44 (16.36)	2,532 (4.23)	0.77
	25-44	256c(34.69)	90,286 (27.45)	528 (28.54)	66,353 (28.75)	784 (30.29)	156,639 (27.99)	95 (35.32)	14,957 (24.96)	0.03
	45-64	169 (22.90)	95,876 (29.15)	305 (16.49)	73,453 (31.83)	474 (18.32)	169,329 (30.26)	58 (21.56)	18595 (31.03)	0.44
	≥65	74 (10.03)	68,805 (20.92)	152 (8.22)	56,458 (24.46)	226 (8.73)	125,263 (22.38)	52 (19.33)	18,588 (31.02)	< 0.05
	Not specified	44 (5.96)	36,558 (11.12)	86 (4.65)	12,857 (5.57)	130 (5.02)	49,415 (8.83)	2 (0.74)	2,981 (4.97)	< 0.05
**Seriousness**										
	Death	13 (1.76)	2,994 (0.91)	17 (0.92)	4,924 (2.13)	30 (1.16)	7,918 (1.41)	1 (0.37)	719 (1.20)	0.30
	Life-threatening	90 (12.20)	3,356 (1.02)	277 (14.97)	4,549 (1.97)	367 (14.18)	7,905 (1.41)	36 (13.38)	789 (1.32)	0.92
	Hospitalization	355 (48.10)	12,328 (3.75)	1,175 (63.51)	25,706 (11.14)	1,530 (59.12)	38,034 (6.80)	131 (48.70)	4,445 (7.42)	< 0.05
	Disability	36 (4.88)	3,682 (1.12)	90 (4.86)	5,615 (2.43)	126 (4.87)	9,297 (1.66)	9 (3.35)	822 (1.37)	0.54
	ER visit	355 (48.10)	37,648 (11.45)	953 (51.51)	31,717 (13.74)	1,308 (50.54)	69,365 (12.39)	149 (55.39)	6,365 (10.62)	< 0.05
**Symptoms**										
	Myocarditis	370 (50.14)	/	1,066 (57.62)	/	1,436 (55.49)	/	136 (50.56)	/	0.12
	Pericarditis	309 (41.87)	/	620 (33.51)	/	929 (35.90)	/	117 (43.49)	/	< 0.05
	Myopericarditis	59 (7.99)	/	164 (8.86)	/	223 (8.62)	/	16 (5.95)	/	0.08
**Manufacturer**										
	Pfizer-BioNTech	407 (55.15)	147,442 (44.83)	1,275 (68.92)	131,427 (56.95)	1,682 (64.99)	278,869 (49.83)	159 (59.11)	30,941 (51.63)	0.05
	Moderna	331 (44.85)	181,439 (55.17)	575 (31.08)	99,354 (43.05)	906 (35.01)	280,793 (50.17)	110 (40.89)	28,987 (48.37)	0.11

VAERS, Vaccine Adverse Event Reporting System; mRNA, messenger RNA; COVID-19, coronavirus disease 2019.

### Disproportionality analysis

The results of overall disproportionality analysis are summarized in **Table 4**. These results revealed significantly high reporting of myocarditis/pericarditis following the administration of both COVID-19 mRNA vaccination programs. Compared with primary series, booster dose of COVID-19 mRNA vaccination did not lead to an increasing risk of myocarditis/pericarditis (primary series: ROR 1.64; 95% CI 1.46–1.83, booster dose: ROR 1.59; 95% CI 1.35–1.86). Among primary series, dose 1 was not associated with signal of myocarditis/pericarditis, while dose 2 was significantly associated with these risks. To further access the individual characteristics, separate sub-analyses based on dose, sex, and age were conducted (**Table 5**). The risks for myocarditis/pericarditis were higher in males when compared with females for all doses. The analysis by age suggested stronger links between the second dose COVID-19 mRNA vaccine and myocarditis/pericarditis among adolescence (ROR 12.91; 95% CI 11.14–14.95) and young adults aged 18 to 24 years (ROR 12.54; 95% CI 10.84–14.52). For dose 1, young adults were significantly associated with the risks of myocarditis/pericarditis with a high ROR of 2.40 (95%CI=1.94–2.97) compared to other age groups. For the booster dose, the risk for myocarditis/pericarditis was highest in young adults aged 18 to 24 years (ROR 6.15; 95% CI 4.48–8.43), and it decreased to 2.80 (95%CI=1.74–4.50) in adolescence. The results showed decreasing association of myocarditis/pericarditis risk with older ages for all doses. To challenge the robustness of the results, sensitivity analyses were performed by comparing PRRs and RORs, which indicated that PRRs for myocarditis/pericarditis were quite similar to RORs for all study sets.

**Table 4:** Results of overall disproportionality analysis.

Vaccination Programs		Pfizer-BioNTech			Moderna			All mRNA		
		Cases	ROR (95%CI)	PRR (95%CI)	Cases	ROR (95%CI)	PRR (95%CI)	Cases	ROR (95%CI)	PRR (95%CI)
**Primary Series**										
	**Dose 1**	407	0.98 (0.85-1.13)	0.98 (0.85-1.13)	331	0.65 (0.55-0.75)	0.65 (0.56-0.75)	738	0.79 (0.70 - 0.90)	0.79 (0.70 - 0.90)
	**Dose 2**	1275	3.43 (3.04-3.87)	3.41 (3.03-3.84)	575	2.05 (1.79-2.34)	2.04 (1.79-2.33)	1850	2.84 (2.53 - 3.18)	2.82 (2.51 - 3.16)
	**In total**	1682	2.13 (1.90-2.40)	2.13 (1.89-2.39)	906	1.14 (1.01-1.29)	1.14 (1.01-1.29)	2588	1.64 (1.46-1.83)	1.63 (1.46-1.83)
**Booster dose**										
	**Dose 3**	159	1.82 (1.51-2.19)	1.81 (1.50-2.19)	110	1.34 (1.08-1.66)	1.34 (1.08-1.66)	269	1.59 (1.35-1.86)	1.59 (1.35-1.86)

ROR, reporting odds ratio; PRR, proportional reporting ratio.

**Table 5:** Sub-group disproportionality analysis of myocarditis/pericarditis following mRNA COVID-19 vaccination compared to all other vaccines from VAERS by age, sex and dose.

Characteristics		Dose 1			Dose 2			Booster dose		
				Cases	ROR (95%CI)	PRR (95%CI)	Cases	ROR (95%CI)	PRR (95%CI)	Cases	ROR (95%CI)	PRR
**Sex**	Female	300	0.46 (0.40–0.54)	0.46 (0.40–0.54)	505	1.14 (1.00–1.31)	1.14 (1.00–1.31)	110	1.04 (0.84–1.28)	1.04 (0.84–1.28)
	Male	427	1.71 (1.49–2.00)	1.71 (1.48–1.97)	1329	6.66 (5.91–7.51)	6.56 (5.82–7.38)	156	2.76 (2.29–3.34)	2.75 (2.28–3.32)
**Age (y)**	<18	81	1.39 (1.09–1.78)	1.39 (1.09–1.77)	387	12.91 (11.14–14.95)	12.49 (10.81–14.42)	18	2.80 (1.74–4.50)	2.78 (1.74–4.46)
	18–24	114	2.40 (1.94–2.97)	2.40 (1.94–2.96)	392	12.54 (10.84–14.52)	12.15 (10.52–14.03)	44	6.15 (4.48–8.43)	6.06 (4.44–8.27)
	25–44	256	1.00 (0.85–1.18)	1.00 (0.85–1.18)	528	2.81 (2.46–3.23)	2.80 (2.44–3.21)	95	2.25 (1.72–2.82)	2.24 (1.78–2.81)
	45–64	169	0.62 (0.52–0.75)	0.62 (0.52–0.75)	305	1.47 (1.26–1.71)	1.47 (1.60–1.71)	58	1.10 (0.83–1.46)	1.10 (0.84–1.46)
	≥65	74	0.38 (0.30–0.49)	0.38 (0.30–0.49)	152	0.95 (0.79–1.15)	0.95 (0.79–1.15)	52	0.99 (0.74–1.33)	0.99 (0.74–1.32)

ROR, reporting odds ratio; PRR, proportional reporting ratio; VAERS, Vaccine Adverse Event Reporting System; mRNA, messenger RNA; COVID-19, coronavirus disease 2019.

### Time to onset of COVID-19 mRNA vaccine related myocarditis/pericarditis

Generally, the median times to event onset of myocarditis/pericarditis was 4 (inter-quartile range [IQR] 1–14) days for dose 1, 3 (IQR 2–17) days for dose 2, and 3 (IQR 1–7) days for dose 3. The times to onset following each dose of COVID-19 mRNA vaccination are shown in **Table 6**. It can be seen from the data that most of the AEFIs of myocarditis/pericarditis occurred within three days after administration of all doses of COVID-19 mRNA vaccination.

**Table 6:** Time to event onset of myocarditis/pericarditis following different doses of mRNA coronavirus 2019 (COVID-19) vaccination.

Onset time (d)	Reports n(%)								
	Pfizer-BioNTech			Moderna			All mRNA		
	Dose 1	Dose 2	Dose 3	Dose 1	Dose 2	Dose 3	Dose 1	Dose 2	Dose 3
0-3	185 (45.45)	706 (55.37)	94 (59.12)	120 (36.25)	283 (49.22)	71 (64.55)	305 (41.33)	989 (53.46)	165 (61.34)
4-7	64 (15.72)	112 (8.78)	21 (13.21)	42 (12.69)	57 (9.91)	22 (20.00)	106 (14.36)	169 (9.14)	43 (15.99)
8-14	52 (12.78)	72 (5.65)	15 (9.43)	35 (10.57)	31 (5.39)	4 (3.64)	87 (11.79)	103 (5.57)	19 (7.06)
>14	72 (17.69)	286 (22.43)	26 (16.35)	81 (24.47)	181 (31.48)	12 (10.91)	153 (20.73)	467 (25.24)	38 (14.13)
Unspecified	34 (8.35)	99 (7.76)	3 (1.89)	53 (16.01)	23 (4.00)	1 (0.91)	87 (11.79)	122 (6.59)	4 (1.49)

## Discussion

To the best of our knowledge, this study is the first real-world population-based study investigating the incidences and risks of myocarditis/pericarditis following booster dose and primary series of COVID-19 mRNA vaccination by assessing reports submitted to the VAERS. This post-marketing safety study stems from recent concerns surrounding whether the booster dose of the COVID-19 mRNA vaccination could lead to an increase in the risks of myocarditis/pericarditis when compared with primary-series course, thus joining the wider debate on the safety of booster-dose COVID-19 vaccination.

COVID-19 mRNA vaccines have been shown to be safe in large-scale trials. Cardiovascular adverse effects in these trials were isolated, with incidences <0.05%, and did not include myocarditis/pericarditis (1, 2). Myocarditis and/or pericarditis has been observed following receipt of mRNA COVID-19 vaccines since their EUAs. According to CDC’s recommendation, people who develop myocarditis or pericarditis after a dose of Moderna, or Pfizer-BioNTech COVID-19 vaccine generally should not receive a subsequent dose of any COVID-19 vaccine (31). Still, there are more cases of myocarditis/pericarditis reported following second dose of mRNA vaccination. ACIP identified that among all the patients with number of vaccine doses received reported, 76% occurred after receipt of dose 2 of mRNA vaccine during December 29, 2020-June 11 (11). Myocarditis/pericarditis rates are ≈ 12.6 cases per million doses of second-dose mRNA vaccine among individuals 12 to 39 years of age, which was significantly higher than that of the first dose (32). The Israeli Ministry of Health also reported 148 myocarditis cases among 10.4 million vaccinated individuals occurring within 30 days of mRNA vaccination, the majority occurring after a second dose (11). Bibhuti and colleagues (33) conducted a cross-sectional study of 29 published cases of acute myopericarditis following COVID-19 mRNA vaccination. The most common presentation in this study was chest pain within 1 to 5 days after the second dose of mRNA COVID-19 vaccination. These facts demonstrated that the risks of myocarditis/pericarditis for second dose of mRNA COVID-19 vaccination are much higher than that of first dose even if people who develop myocarditis or pericarditis after the first dose might not receive a second dose according to CDC’s recommendation. Correspondingly, deep concerns about whether the booster dose could lead to an increase in the risks of myocarditis/pericarditis have been raised.

This study used the VAERS to retrieve reports of myocarditis/pericarditis following COVID-19 mRNA vaccination and the CDC COVID Data tracker to measure the number of vaccinated people or the total doses of administered COVID-19 mRNA vaccines during the same period. Hence, the common limitation of the passive surveillance data of unknown denominators was solved. Our results indicate that myocarditis/pericarditis represented rare AEFIs for both primary-series and booster-dose COVID-19 mRNA vaccinations. In the general US population, the incidence rates of myocarditis and pericarditis are 5.73 to 26 cases per 100,000 person-year and 0.95 to 2.16 cases per 100,000 person-year, respectively (34). Compared with the general population, our study showed higher incidences of myocarditis and pericarditis after administration of COVID-19 mRNA vaccination, which is consistent with the result of Li’s previous study (35). Of note, Li only estimated cases from the primary vaccine series; thus, the study did not provide safety evidence for the booster-dose vaccination. Our results show that the incidence rate of myocarditis/pericarditis following booster dose is significantly lower than that after primary-series vaccination. Historically, the occurrence of myocarditis/pericarditis following vaccinations is not new. Rare occurrences of myocarditis/pericarditis were found to be associated with smallpox vaccination, hepatitis B, or other vaccines (18). Another surveillance study identified an incidence rate of 5.5 per million vaccine recipients for myocarditis/pericarditis following the smallpox vaccination (36). Despite significantly high incidence of myocarditis/pericarditis following the second dose of mRNA vaccines, the incidences after the first and booster doses of the mRNA COVID-19 vaccination are lower than that following smallpox vaccines.

Data mining based on disproportionality analyses within VAERS has been widely used to detect safety signals of vaccines. Signals for inactivated influenza and typhoid and tetanus toxoid-containing vaccines have been successfully identified as described in previous studies (37, 38). An earlier disproportionality study (35) found an increased risk for myocariditis/pericarditis following primary-series mRNA COVID-19 vaccines. The result is consistent with ACIP’s conclusion that a likely association between COVID-19 mRNA vaccines and cases of myocarditis and pericarditis exists (11). A relevant benefit–risk assessment was conducted by ACIP thereafter, which concluded that the benefits outweigh the risks, and continued use of mRNA COVID-19 vaccines is recommended in all age groups (11). It should be noted that the assessment was conducted before the recommendation of booster dose. Thus, to further explore the cardiac safety of booster-dose mRNA COVID-19 vaccination, a comprehensive analysis was conducted by comparing the disproportionalities between primary series and booster dose. The results showed that the ROR and PRR of booster doses are lower than that of primary series, indicating that booster shots of mRNA COVID-19 vaccination does not increase the risk of myocarditis/pericarditis.

Two-thousand five-hundred eighty-eight reports of myocarditis/pericarditis following primary-series mRNA COVID-19 vaccination in VAERS during the analytic period were found, 71.48% of which occurred following the second dose. This result is consistent with the findings of published cases with reports predominantly after the second dose of mRNA COVID-19 vaccines (39–41). In addition, a separate subgroup disproportionality analysis based on the dose administered was conducted. Dose 1 showed a lower ROR of 0.79 (95% CI: 0.70–0.90), demonstrating that the first dose was not associated with any signs of myocarditis/pericarditis. Not surprisingly, the results revealed significantly high reporting of myocarditis/pericarditis following the administration of the second dose. The disproportionality results of ROR and PRR for dose 3 were both lower than dose 2, further confirming the cardiac safety of the booster dose.

Males accounted for most of the cases of myocarditis/pericarditis following both primary and booster shots of mRNA COVID-19 vaccines. Sub-group disproportionality analysis also indicated that the risks for myocarditis/pericarditis were higher in males when compared with females for all doses. Male predominance in myocarditis/pericarditis cases has previously been described in the general population and postvaccination of mRNA COVID-19 vaccines (33, 35, 42, 43). The specific mechanism for this phenomenon is still unknown. Sex hormone differences are thought to be an important reason for this difference (39, 44, 45). Estrogen could inhibit pro-inflammatory T-cells, resulting in a decrease in cell-mediated immune responses, while testosterone has inhibitory effects on anti-inflammatory cells (32). Published studies have also indicated higher incidence of myocarditis/pericarditis in adolescents following the administration of the second dose of mRNA COVID-19 vaccines (11). The adolescent predominance for dose 2 has been verified with a high ROR of 12.91 in our study. Surprisingly, the risks were higher in young adults aged 18–44 years for dose 1 and booster dose. Thorough investigations are needed to clarify the differences of age predominance between doses. Patients in anecdotal reports usually present with myocarditis-associated symptoms 2–3 days after mRNA COVID-19 vaccination (7, 46, 47). The median time to the onset of myocarditis/pericarditis was 3 (IQR 1–7) days following booster shot of mRNA COVID-19 vaccine, which was similar to the time to onset reported for doses 1 and 2.

Several hypotheses have been proposed for the potential mechanisms of mRNA COVID-19 vaccination-induced myocarditis/pericarditis (32, 48). mRNA COVID-19 vaccines contain nucleoside-modified mRNA encoding the viral spike glycoprotein of COVID-19 that will produce an immune response in the recipient (49). In theory, nucleoside modifications of mRNA could lead to a reduction in innate immunogenicity and result in less cytokine activation. COVID-19 mRNA vaccines have been shown to be safe based on data from large-scale trials (1, 2). While in certain vaccine recipients with genetic predisposition, the innate immunogenicity may not be scaled back and result in the activation of an aberrant immune response (50, 51). Another study found that antibodies against COVID-19 spike glycoproteins could cross-react with structurally similar human peptide protein sequences, such as α-myosin (52), making the molecular mimicry between the self-antigens and the spike protein of COVID-19 another possible mechanism for mRNA COVID-19 vaccination associated myocarditis/pericarditis. Hajjo and colleagues (45) conducted an informatics study with systems biology methods to explore the potential mechanisms for development of myocarditis/pericarditis. The results suggest a central role of interferon-gamma (INF-γ) signaling in the biological processes leading to cardiac AEs, which can be modulated by increasing the time interval between doses. Thus, this time factor could be part of the reason for second dose predominance in myocarditis/pericarditis since the time interval between doses 2 and 3 are longer than that of doses 1 and 2.

Study limitations should be acknowledged. These limitations are mainly inherent to the nature of self-reporting database (23). First, cases in VAERS might contain information that is incomplete and inaccurate, especially the lack of information on concomitant medications or comorbid medical histories. However, vaccine safety experts review all reports of serious AEs and perform further investigations for confirmation if needed. For example, all adults are given the same dose of vaccine regardless of their body weight. The raw data of VAERSDATA doesn’t contain content regarding body weight information. Therefore, dose dependent sub-analysis could be conducted to assessing dose/kg how it correlates to myocarditis/pericarditis. Second, AEs are usually under-reported in VAERS, which may lead to an underestimation of the actual associated risks. However, serious AEs, such as myocarditis/pericarditis, examined in this study are more likely to be reported than nonserious ones (35). Last, all reports are submitted to VAERS without assessing specific causality considering the events may be coincidental and related to other causes. This type of reporting might lead to an overestimation of the associated risks. Notwithstanding these limitations, disproportionality analysis still represents an invaluable method for monitoring vaccine safety and identifying novel rare signals.

In conclusion, our study showed that the booster dose of COVID-19 mRNA vaccination when compared with primary series course does not lead to an increase in the risks of myocarditis/pericarditis. The risks for myocarditis/pericarditis were higher in males when compared with females for all doses. Among the primary series, the second dose was associated with risks of myocarditis/pericarditis, while the first dose was not. The risks for myocarditis/pericarditis were highest in young adults aged 18 to 24 years for booster dose and in adolescence for the second dose. Most of the AEFIs of myocarditis/pericarditis occurred within three days after administration of all doses of COVID-19 mRNA vaccination.

## Data availability statement

The original contributions presented in the study are included in the article/supplementary materials. Further inquiries can be directed to the corresponding authors.

## Ethics statement

This pharmacovigilance study used publicly available, deidentified data. Ethical review and approval was not required for the study on human participants in accordance with the local legislation and institutional requirements. Written informed consent from the participants’ legal guardian/next of kin was not required to participate in this study in accordance with the national legislation and the institutional requirements.

## Author contributions

JF and JX conceived and designed this study. CC contributed to data analysis and interpretation, wrote, and revised the manuscript. FF and LD contributed to data analysis and revised the manuscript. All authors contributed to the article and approved the submitted version.

## Funding

This work was partially supported by The Medical and Health Guidance Project of Xiamen (3502Z214ZD1168).

## Acknowledgments

We thank Xiaoshan Yu, a pharmacovigilance specialist, for her role in reviewing disproportionality analysis.

## Conflict of interest

The authors declare that the research was conducted in the absence of any commercial or financial relationships that could be construed as a potential conflict of interest.

## Publisher’s note

All claims expressed in this article are solely those of the authors and do not necessarily represent those of their affiliated organizations, or those of the publisher, the editors and the reviewers. Any product that may be evaluated in this article, or claim that may be made by its manufacturer, is not guaranteed or endorsed by the publisher.
